# Metabolic obesity phenotypes and obesity‐related cancer risk in the National Health and Nutrition Examination Survey

**DOI:** 10.1002/edm2.433

**Published:** 2023-06-05

**Authors:** Maci Winn, Prasoona Karra, Heinz Freisling, Marc J. Gunter, Benjamin Haaland, Michelle L. Litchman, Jennifer A. Doherty, Mary C. Playdon, Sheetal Hardikar

**Affiliations:** ^1^ Department of Population Health Sciences University of Utah Salt Lake City Utah USA; ^2^ Huntsman Cancer Institute University of Utah Salt Lake City Utah USA; ^3^ Department of Nutrition and Integrative Physiology University of Utah Salt Lake City Utah USA; ^4^ Nutrition and Metabolism Branch International Agency for Research on Cancer Lyon France; ^5^ University of Utah College of Nursing Salt Lake City Utah USA; ^6^ Fred Hutchinson Cancer Research Center Seattle Washington USA

**Keywords:** cancer risk, epidemiology, metabolic obesity phenotypes, metabolic syndrome, obesity‐related cancer

## Abstract

**Introduction:**

Body mass index (BMI) fails to identify up to one‐third of normal weight individuals with metabolic dysfunction who may be at increased risk of obesity‐related cancer (ORC). Metabolic obesity phenotypes, an alternate metric to assess metabolic dysfunction with or without obesity, were evaluated for association with ORC risk.

**Methods:**

National Health and Nutrition Examination Survey participants from 1999 to 2018 (*N* = 19,500) were categorized into phenotypes according to the metabolic syndrome (MetS) criteria and BMI: metabolically healthy normal weight (MHNW), metabolically unhealthy normal weight (MUNW), metabolically healthy overweight/obese (MHO) and metabolically unhealthy overweight/obese (MUO). Adjusted multivariable logistic regression models were used to evaluate associations with ORC.

**Results:**

With metabolic dysfunction defined as ≥1 MetS criteria, ORC cases (*n* = 528) had higher proportions of MUNW (28.2% vs. 17.4%) and MUO (62.6% vs. 60.9%) phenotypes than cancer‐free individuals (*n* = 18,972). Compared with MHNW participants, MUNW participants had a 2.2‐times higher ORC risk [OR (95%CI) = 2.21 (1.27–3.85)]. MHO and MUO participants demonstrated a 43% and 56% increased ORC risk, respectively, compared to MHNW, but these did not reach statistical significance [OR (95% CI) = 1.43 (0.46–4.42), 1.56 (0.91–2.67), respectively]. Hyperglycaemia, hypertension and central obesity were all independently associated with higher ORC risk compared to MHNW.

**Conclusions:**

MUNW participants have a higher risk of ORC than other abnormal phenotypes, compared with MHNW participants. Incorporating metabolic health measures in addition to assessing BMI may improve ORC risk stratification. Further research on the relationship between metabolic dysfunction and ORC is warranted.

## Graphical abstract

Participants in the National Health and Nutrition Examination Survey who were metabolically unhealthy but had a normal body weight have a higher risk of obesity‐related cancer compared to participants who are metabolically healthy (absence of hyperglycaemia, hypertension, dyslipidaemia and central adiposity). These observations suggest that tools such as metabolic obesity phenotypes may be a better method of risk stratifying individuals for ORC than obesity measures alone.

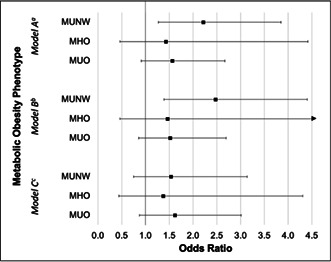

## 1 INTRODUCTION

Over 40% of the United States (US) population is obese, and this proportion is expected to increase as the obesity pandemic escalates.^1^ Obesity has been linked to the risk of multiple comorbid conditions, including Type II diabetes, cardiovascular disease and cancer.^2^ The National Cancer Institute and International Agency for Research on Cancer has categorized the following 13 cancers as having a causal relationship with obesity: breast (postmenopausal), colorectal, uterine, ovarian, pancreatic, liver, gallbladder, kidney (renal cell), thyroid, multiple myeloma, meningioma, oesophageal adenocarcinoma and gastric cardia cancer.^2^ The incidence of these obesity‐related cancers (ORC) has been increasing in concert with obesity.^3^ Although chronic inflammation, alterations in hormone signalling, hyperinsulinemia and hyperglycaemia, dyslipidaemia and oxidative stress have been proposed as mechanisms involved in the aetiology of ORC, the precise mechanisms are largely unknown.^2^, ^4^, ^5^, ^6^, ^7^

Body mass index (BMI), the clinical standard for measuring obesity, fails to identify up to a third of normal weight individuals with metabolic dysfunction common to obesity.^8^ As an alternative to examining body mass or metabolic health status alone, metabolic obesity phenotype is an emerging concept that characterizes the status of metabolic dysfunction among individuals with or without overweight or obesity according to BMI. Metabolic dysfunction is often defined using metabolic syndrome (MetS) criteria or its components, but criteria have differed across studies.^9^ The four commonly defined metabolic obesity phenotypes include: metabolically healthy normal weight (MHNW), metabolically unhealthy normal weight (MUNW), metabolically healthy overweight/obese (MHO) and metabolically unhealthy overweight/obese (MUO). Although metabolic obesity phenotypes have been studied in relation to multiple cardiometabolic diseases, fewer studies have examined ORC risk as an outcome, particularly in the United States.^6^, ^10^, ^11^, ^12^, ^13^, ^14^, ^15^, ^16^, ^17^, ^18^, ^19^, ^20^, ^21^, ^22^, ^23^ These studies have observed associations between the metabolically unhealthy phenotypes and risk of individual cancer types, either with or independent from obesity. However, further research is needed to determine the strength and direction of these associations, while accounting for different definitions of metabolic dysfunction.

The goal of this study was to determine the prevalence of metabolic obesity phenotypes by ORC status, and to investigate the risk of ORC associated with the different metabolic obesity phenotypes.

## 2 METHODS

### 2.1 Participant population

The current study used data from the National Health and Nutrition Examination Survey (NHANES).^24^ At 2‐year intervals, NHANES interviews approximately 7000 unique participants, who complete questionnaires, a physical exam and provide biospecimens for biomarker measurement. All participants provide written informed consent, and study protocols are approved by the Institutional Review Boards at the Centers for Disease Control and Prevention. These data are made publicly available and are therefore exempt from institutional review. All participants ≥18 years who provided a fasting blood sample between 1999 and 2018 were considered for inclusion in our analysis. Participants with fasting hypoglycaemia (fasting blood glucose <70 mg/dL), with ≥3 missing values for the MetS criteria (including hyperglycaemia, hypertension, low high‐density lipoprotein [HDL]‐cholesterol, hypertriglyceridaemia and central obesity classified by waist circumference), with a cancer other than ORC, or who were breastfeeding or pregnant were excluded.

### 2.2 Exposures, confounding variables and outcomes

The metabolic obesity phenotypes were defined according to BMI [normal weight: BMI <25 kg/m^2^; overweight/obese: BMI ≥25 kg/m^2^] and the MetS criteria defined by the National Cholesterol Education Program's Adult Treatment Panel III (NCEP ATPIII) [hyperglycaemia (fasting blood glucose ≥100 mg/dL), hypertension (diastolic blood pressure ≥85 mmHg or systolic blood pressure ≥130 mmHg), abdominal obesity (waist circumference >88 cm (female) or >102 cm (male)), elevated triglycerides (≥150 mg/dL) and low HDL‐cholesterol (<50 mg/dL (female) or <40 mg/dL (male)) or drug treatment for these parameters].^25^ Three distinct definitions of metabolic dysfunction were used to compute three separate metabolic obesity phenotype variables: (1) a broad definition of metabolic dysfunction, categorising participants with ≥1 MetS criteria as metabolically unhealthy; (2) participants with mild metabolic dysfunction, that is, categorising participants with 1–2 MetS criteria as metabolically unhealthy (participants with 3–5 MetS criteria were excluded from this analysis); and (3) the traditional clinical definition of MetS, categorising participants with ≥3 MetS criteria as metabolically unhealthy (participants with 1–2 MetS criteria were excluded from this analysis). Investigating multiple definitions of metabolic dysfunction allowed us to examine both mild dysfunction (individuals with only 1–2 MetS criteria) as well as the strict clinically used definition of metabolic dysfunction (≥3 MetS criteria). All models were analysed with MHNW as the reference group, and metabolically healthy was defined as zero abnormal MetS parameters.

The NHANES medical conditions questionnaires were used to identify participants with an ORC. ORCs included breast, colorectal, uterine, ovarian, pancreatic, liver, gallbladder, kidney and thyroid cancer.^2^ As NHANES lacks the histologic information needed to determine obesity‐related subtypes of oesophageal, gastric, blood and brain cancers (*n* = 107), these cancers were excluded from our analysis. Presence of ORC was established on two questionnaire items: (1) “Have you ever been told by a doctor or other health professional that you had cancer or a malignancy of any kind?”; and (2) “What kind of cancer?”

Statistical analyses were adjusted for age (<50, 50–59, 60–69, 70–79, ≥80 years), sex, race/ethnicity [non‐Hispanic White, non‐Hispanic Black, Mexican American/Other Hispanic and Other (including non‐Hispanic Asian and all non‐Hispanic persons that reported races other than Black, Asian or White)], highest education level completed (grade school, high school, college, missing), yearly household income (<$35,000, $35,000–$74,999, ≥$75,000, missing), smoking status (never, former, current, missing), alcohol use (yes, no, missing), daily hours sedentary (daily hours available 2007–2018; daily computer, video game and TV hours used for 1999–2006, missing), weekly physical activity (low/no, moderate, vigorous, missing), average daily caloric intake (using 2 days of dietary recall data) and survey year.

### 2.3 Statistical analysis

Descriptive statistics for ORC and cancer‐free participants were computed. Adjusted multivariable logistic regression models were used to compute odds ratios (OR) and 95% confidence intervals (CI) for risk of ORC with each metabolic obesity phenotype compared to MUNW participants. These models were adjusted for age and sex, and then separately adjusted for all covariates outlined above.

Next, we performed exploratory analyses to identify individual components of MetS that may contribute more strongly to the association between “unhealthy” metabolic phenotype status and risk of ORC, compared to MHNW individuals. We constructed separate models where; (1) the “metabolically unhealthy” definition included all participants with an abnormal value for the metabolic parameter of interest (these participants may have abnormal values for other parameters, in addition to the parameter of interest); and (2) only participants with the single abnormal parameter of interest, were analysed (participants with abnormal parameters other than the one of interest were excluded).

Multiple sensitivity analyses were performed: (1) excluding waist circumference from the metabolically unhealthy definition due to the high correlation with BMI (*r* ^2^ = 0.90, *p* < .0001); (2) adjusting for menopausal status and hormone replacement therapy (HRT) use in female participants as several ORCs are cancers of the female reproductive system, and obesity is particularly associated with postmenopausal breast cancer; (3) using obesity defined as ≥30 kg/m^2^ (vs. with 25 ≤ BMI <30 kg/m^2^) to evaluate metabolic obesity phenotype characterized by severe adiposity; and (4) complete case analysis excluding all participants with missing values for any of the exposures.

All estimates were weighted in accordance with the NHANES analytic guidelines to be representative of the U.S. civilian noninstitutionalized resident population.^26^ Fasting blood sample weights “wtsaf4yr” (4‐year fasting weights for 1999–2002 NHANES participants) and “wtsaf2yr” (2‐year fasting weights for 2003–2018 NHANES participants) were added to all statistical models because our primary exposure, metabolic obesity phenotypes, was computed using the MetS criteria and includes multiple biomarker measurements. NHANES recommends the weights “wtsaf4yr” and “wtsaf2yr” to be included when analysing the biomarker data. Implausible measurements (BMI >130 kg/m^2^, diastolic blood pressures <40 mmHg) were changed to missing. All analyses were performed using SAS version 9.4 and significance was determined at *α* = 0.05.

## 3 RESULTS

We evaluated associations of metabolic obesity phenotypes with ORC among NHANES participants from 1999 to 2018. Descriptive statistics by cancer status are outlined in Table 1, and descriptive statistics by metabolic obesity phenotype are outlined in Table S1. Overall, 19,500 NHANES participants were included in the analyses, of whom 18,972 were cancer‐free while 528 (2.7%) reported a previous history of ORC. Participants with ORC were older (16.5% vs. 3.0% aged ≥80 years), female (90.4% vs. 50.0%) and non‐Hispanic White (78.9% vs. 67.1%). Female participants with ORC were more likely to be postmenopausal (87.0% vs. 45.1%) and more likely to have used HRT (39.7% vs. 20.5%), compared with cancer‐free participants. Metabolically unhealthy participants were more likely to be older, postmenopausal, use HRT and have a lower physical activity level compared with metabolically healthy participants. MHNW participants were more likely to be female, while MHO participants were more likely to be male. Additionally, we observed an increasing trend with BMI and metabolic obesity phenotype (mean BMI: MHNW, 21.8; MUNW, 22.4; MHO, 27.3; MUO, 32.1). There were no differences observed by other characteristics.

**TABLE 1:** Demographic information for National Health and Nutrition Examination Survey (NHANES) participants with a self‐report prior history of obesity‐related cancer and participants without a prior history of cancer (*N* = 19,500).

	Cancer‐free, *n* = 18,972			ORC cases, *n* = 528		
	Actual frequency	Weighted frequency/mean	%/SE	Actual frequency	Weighted frequency/mean	%/SE
*Age in years* ^a^						
<50	10,772	111,661,231	62.4	72	880,275	19.3
50–59	2785	30,898,488	17.3	70	657,088	14.4
60–69	2819	20,554,320	11.5	136	1,156,191	25.3
70–79	1643	10,614,122	5.9	143	1,120,230	24.5
≥80	953	5,322,227	3.0	107	752,739	16.5
*Sex*						
Female	9418	89,438,085	50.0	458	4,129,970	90.4
Male	9554	89,612,304	50.0	70	436,554	9.6
*Race/ethnicity* ^b^						
White (non‐Hispanic)	8013	120,085,880	67.1	313	3,604,119	78.9
Black (non‐Hispanic)	3954	21,175,626	11.8	83	399,216	8.7
MA/Hispanic	5402	25,572,541	14.3	108	387,766	8.5
Other	1603	12,216,343	6.8	24	175,422	3.8
*Body mass index (kg/m* ^*2*^ *)* ^d^						
<25 (normal weight)	6037	58,470,916	32.7	153	1,537,118	33.7
25–<30 (overweight)	6221	58,409,554	32.6	160	1,321,814	28.9
≥30 (obese)	6442	60,037,748	33.5	205	1,608,274	35.2
*Smoking* ^d^						
Never	9701	93,390,439	52.2	284	2,356,639	51.6
Former	4219	41,489,739	23.2	180	1,585,134	34.7
Current	3769	38,566,999	21.5	63	622,797	13.6
*Alcohol use* ^d^						
No	4317	35,235,451	19.7	191	1,526,857	33.4
Yes	10,467	109,844,930	61.3	254	2,310,722	50.6
*Physical activity* ^d^						
No/low activity	6567	52,576,085	29.4	243	1,771,792	38.8
Moderate/vigorous activity	12,123	124,284,587	69.4	263	2,630,271	57.6
*Sedentary* ^d^						
<5 h	10,914	101,351,152	56.6	284	2,446,702	53.6
≥5 h	8052	77,658,500	43.4	244	2,119,822	46.4
Total daily caloric intake^d^		2158	9.1		1701	29.4
*Menopausal status* ^c^ ^,^ ^d^						
Premenopausal	4110	42,330,530	47.3	33	342,598	8.3
Postmenopausal	4509	40,352,542	45.1	397	3,591,571	87.0
*HRT use* ^c^ ^,^ ^d^						
No	6190	61,115,286	68.3	270	2,269,323	54.9
Yes	1746	18,338,752	20.5	157	1,641,336	39.7
*Income* ^d^						
<$35,000	8955	66,673,797	37.2	267	1,962,443	43.0
$35,000–$75,000	5498	57,438,589	32.1	153	1,483,172	32.5
>$75,000	2479	32,259,400	18.0	59	599,775	13.1
*Education* ^d^						
<High school	5538	34,493,851	19.3	155	947,800	20.8
High school	4488	43,168,833	24.1	125	1,264,076	27.7
>High school	8922	101,213,672	56.5	248	2,354,648	51.6

Abbreviations: HRT, hormone replacement therapy; MA, Mexican American.

^a^ Participants with age ≥ 85 are recorded as age = 85 in the NHANES data sets.

^b^ The “other” race category in NHANES includes non‐Hispanic Asian and all non‐Hispanic persons that reported races other than Black, Asian or White.

^c^ Among female study participants only.

^d^ Data was missing for the following: income: 10.7%, education: 0.1%, smoking: 6.6%, BMI: 1.4%, physical activity: 1.6%, sedentary behaviour: 0.0%, alcohol: 21.9%, total daily caloric intake: 3.5%, menopausal status: 8.4%, HRT: 15.3%.

**FIGURE 1:**
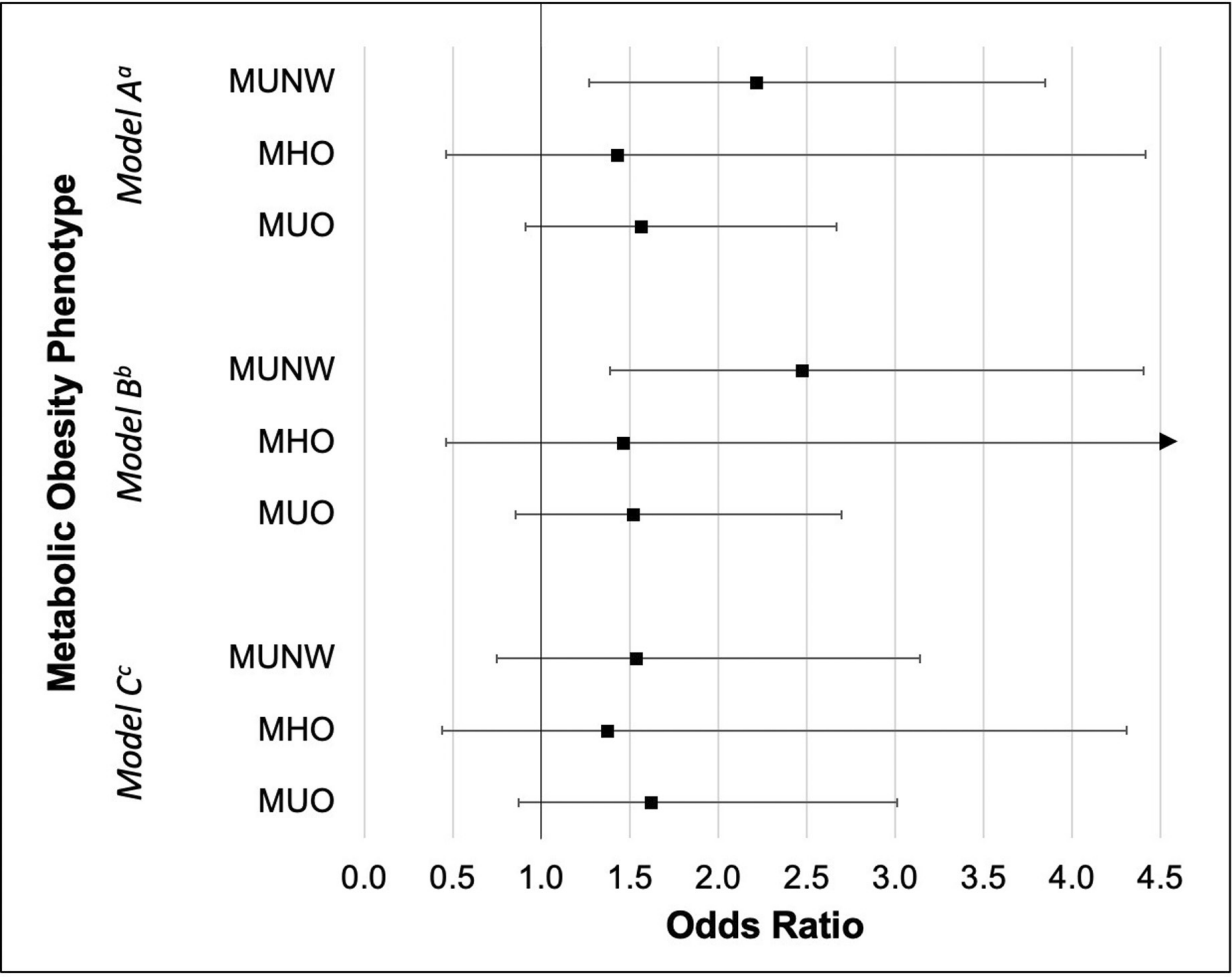
Adjusted odds ratios and 95% confidence intervals for risk of obesity‐related cancer by metabolic obesity phenotype, compared to metabolically healthy normal weight participants in the National Health and Nutrition Examination Survey (NHANES) 1999–2018 (*N* = 19,500)^d^. ^a^Model A: metabolic dysfunction defined as one or more metabolic syndrome (MetS) criteria. ^b^Model B: metabolic dysfunction defined as one or two MetS criteria. ^c^Model C: metabolic dysfunction as the clinical definition of MetS (3 or more MetS criteria). ^d^All analyses were adjusted for age, sex, race/ethnicity, education level, annual household income, smoking status, alcohol use, daily hours sedentary, weekly physical activity level, daily calorie intake and survey year. MUNW, metabolically unhealthy normal weight; MHO, metabolically healthy overweight/obese; MUO, metabolically unhealthy overweight/obese.

Metabolic obesity phenotype analyses are displayed in Table 2. Three separate models with distinct definitions of “metabolically unhealthy” were computed to determine the association of different levels of metabolic dysfunction with ORC risk (Figure 1). In *Model A*, metabolic dysfunction was defined more broadly as presence of one or more of the five MetS criteria. Using this definition, MUNW individuals had 2.2‐times higher risk of ORC compared to MHNW individuals [OR (95% CI): 2.21 (1.27–3.85)]. MHO and MUO individuals had a 43% and 56% higher risk of ORC, respectively, but these did not reach statistical significance [OR (95% CI): 1.43 (0.46–4.42) and 1.56 (0.91–2.67), respectively]. In *Model B*, metabolic dysfunction was defined as presence of only one or two MetS criteria (participants with ≥3 criteria were excluded from this analysis). Compared with MHNW individuals, participants who were MUNW had 2.5‐times higher risk of ORC [OR(95% CI): 2.48 (1.39–4.41)]. Similarly, MHO and MUO participants also had a higher risk of ORC, but these did not reach statistical significance. *Model C* included the strictest definition of metabolic dysfunction, where metabolic dysfunction was defined as the clinical definition of MetS (i.e. three or more criteria). Using this strict definition, no abnormal metabolic obesity phenotype was associated with ORC risk compared to MHNW.

**TABLE 2:** Adjusted odds ratios (OR) and 95% confidence intervals (CI) for obesity‐related cancer (ORC) by each metabolic obesity phenotype in National Health and Nutrition Examination Survey participants (*N* = 19,500).

Metabolic obesity phenotype	Cancer‐free		ORC cases		Age and sex adjusted		Fully adjusted^a^	
	*N* (actual)	% (weighted)	*N* (actual)	% (weighted)	OR	95% CI	OR	95% CI
*Metabolic dysfunction defined as one or more MetS criteria (Model A)* ^b^								
MHNW (Ref)	2584	15.2	21	5.5	REF	REF	REF	REF
MUNW	3453	17.4	132	28.2	2.18	1.25–3.81	2.21	1.27–3.85
MHO	921	5.2	7	1.6	1.42	0.47–4.28	1.43	0.46–4.42
MUO	11,742	60.9	358	62.6	1.61	0.93–2.77	1.56	0.91–2.67
*Metabolic dysfunction defined as one or two MetS criteria (Model B)* ^c^								
MHNW (Ref)	2584	22.6	21	9.9	REF	REF	REF	REF
MUNW	2871	21.9	101	41.1	2.39	1.35–4.25	2.48	1.39–4.41
MHO	921	7.7	7	2.9	1.45	0.48–4.41	1.46	0.46–4.63
MUO	5786	46.1	120	42.2	1.50	0.85–2.64	1.52	0.85–2.70
*Metabolic dysfunction defined as the clinical definition of MetS (≥3 MetS criteria) (Model C)* ^d^								
MHNW (Ref)	2584	28.1	21	10.2	REF	REF	REF	REF
MUNW	582	5.0	31	10.0	1.50	0.73–3.10	1.53	0.75–3.14
MHO	921	9.6	7	2.9	1.38	0.45–4.21	1.38	0.44–4.31
MUO	5956	55.1	238	72.8	1.67	0.88–3.16	1.62	0.87–3.01

Abbreviations: MetS, metabolic syndrome; MUNW, metabolically unhealthy normal weight; MHO, metabolically healthy overweight/obese; MUO, metabolically unhealthy overweight/obese.

^a^ Adjusted for age, sex, race/ethnicity, education level, annual household income, smoking status, alcohol use, daily hours sedentary, weekly physical activity level, daily calorie intake and survey year.

^b^ Model A: cancer‐free, *n* = 18,972; ORC cases, *n* = 528.

^c^ Model B: cancer‐free, *n* = 12,434; ORC cases, *n* = 259 (excludes participants with ≥3 MetS criteria).

^d^ Model C: cancer‐free, *n* = 10,315; ORC cases, *n* = 307 (excludes participants with 1–2 MetS criteria).

To evaluate which of the metabolic dysfunction parameters were driving the increased risk for ORC, additional exploratory analyses were performed for each MetS criteria individually as the metabolic dysfunction component of the metabolic obesity phenotype (Table 3). Compared with MHNW participants, MUNW participants with hyperglycaemia (not excluding participants with other abnormal MetS parameters) had a 1.6‐times higher risk of ORC [OR (95% CI): 1.63 (1.05–2.52)]. MUO participants with only hypertension or only central obesity (other MetS parameters within clinical range) had a 3.7‐ and 2.2‐times higher risk of ORC, respectively, compared to MHNW participants [OR (95% CI): 3.65 (1.33–10.00) and 2.19 (1.01–4.77), respectively].

**TABLE 3:** Adjusted odds ratios (OR) and 95% confidence intervals (CI) for obesity‐related cancer (ORC) by each metabolic obesity phenotype, using individual parameter definitions of metabolically unhealthy, in National Health and Nutrition Examination Survey participants (*N* = 19,500).

“Unhealthy definition”	All participants with parameter of interest						Only participants with single abnormal parameter					
	Cancer‐free		ORC cases		OR	95% CI	Cancer‐free		ORC cases		OR	95% CI
	*N* ^a^	%^b^	*N* ^a^	%^b^			*N* ^a^	%^b^	*N* ^a^	%^b^		
*Hyperglycaemia*												
MHNW (Ref)	4255	24.3	83	18.9	REF	REF	2584	56.6	21	47.5	REF	REF
MUNW	1782	8.3	70	14.7	1.63	1.05–2.52	683	13.0	16	25.9	2.35	1.00–5.51
MHO	5740	31.7	113	22.6	0.94	0.62–1.42	921	19.4	7	13.8	1.38	0.36–5.32
MUO	6923	34.4	252	41.5	1.07	0.79–1.44	474	9.6	5	8.3	1.63	0.55–4.80
*Low‐HDL*												
MHNW (Ref)	4321	23.5	108	24.8	REF	REF	2584	63.4	21	72.3	REF	REF
MUNW	1027	5.4	26	4.7	0.71	0.45–1.13	377	8.6	3	6.7	0.61	0.17–2.21
MHO	6837	35.6	188	33.6	0.77	0.56–1.06	921	21.8	7	21.0	1.16	0.27–4.96
MUO	4556	23.7	140	23.4	0.82	0.57–1.19	214	5.0	N/A	N/A	N/A	N/A
*Hypertriglyceridaemia*												
MHNW (Ref)	4985	27.3	110	24.6	REF	REF	2584	66.2	21	61.7	REF	REF
MUNW	975	5.0	38	7.7	1.08	0.64–1.82	203	5.3	4	14.0	2.19	0.37–12.97
MHO	8050	41.5	201	33.9	0.78	0.57–1.08	921	22.7	7	17.9	1.39	0.34–5.68
MUO	4498	24.1	159	29.4	1.04	0.70–1.56	170	4.5	3	6.4	1.96	0.21–17.90
*Hypertension*												
MHNW (Ref)	4471	25.5	66	15.9	REF	REF	2584	61.8	21	35.2	REF	REF
MUNW	1561	7.1	87	17.8	1.32	0.83–2.09	564	10.7	29	37.9	1.85	0.76–4.54
MHO	7300	40.9	149	29.8	1.00	0.67–1.49	921	21.2	7	10.2	1.33	0.39–4.50
MUO	5358	25.2	216	34.4	0.93	0.63–1.38	281	5.0	11	13.7	3.65	1.33–10.00
*Central obesity*												
MHNW (Ref)	5505	29.7	115	24.9	REF	REF	2584	52.3	21	36.0	REF	REF
MUNW	423	2.5	35	8.1	1.17	0.67–2.06	100	2.6	5	9.2	2.20	0.58–8.35
MHO	3305	17.2	24	4.7	0.72	0.39–1.34	921	17.9	7	10.4	1.50	0.40–5.67
MUO	9085	47.8	320	55.1	0.84	0.62–1.15	1331	26.3	28	44.3	2.19	1.01–4.77

Abbreviations: HDL, high density lipoprotein‐cholesterol; MUNW, metabolically unhealthy normal weight; MHO, metabolically healthy overweight/obese; MUO, metabolically unhealthy overweight/obese.

^a^ Actual frequency.

^b^ Weighted percentage.

The results for sensitivity analyses excluding waist circumference from the metabolically unhealthy definition, adjusting for menopausal status and HRT use, defining obesity as ≥30 kg/m^2^, and the complete case analysis are displayed in Tables S2–S5. Results for these analyses were similar in magnitude and direction to our primary analyses.

## 4 DISCUSSION

In this study, we investigated the associations between metabolic obesity phenotypes and risk of ORC in 19,500 NHANES participants and observed a higher ORC risk in participants who were metabolically unhealthy but had a normal weight compared with MHNW participants. Although the current clinical definition of MetS categorizes individuals with ≥3 abnormal parameters as being metabolically unhealthy, our results suggest that even mild metabolic dysfunction might be associated with ORC risk in normal weight individuals. Thus, tools such as metabolic obesity phenotypes may be useful in better identifying individuals at a higher risk of ORC, beyond just BMI alone.

Although evidence supporting a relationship between metabolic dysfunction and cancer risk has been increasing,^27^, ^28^, ^29^ the mechanisms are still poorly understood. The most commonly hypothesized mechanism is chronic inflammation resulting from excess adipose tissue, including immune cell infiltration, and cytokine and chemokine release that creates a preneoplastic microenvironment.^4^, ^30^, ^31^ Insulin resistance, another marker of metabolic dysfunction, has been independently linked to ORC risk,^31^ and has been shown to interact synergistically with inflammation in carcinogenesis.^31^ Although insulin resistance is well known to be associated with visceral obesity, it has also been seen in individuals with a normal BMI.^32^ Alterations in the gut microbiome may contribute to chronic inflammation, insulin resistance and increased cancer risk independent of obesity.^33^

Individuals with excess fat and an increased waist circumference may not always be classified as obese when BMI alone is used as an obesity metric, and therefore may not be perceived to have an increased cancer risk. Indeed, in our data set, 5.2% of participants with an elevated waist circumference had a normal BMI. Our results show that participants with the MUNW phenotype have the highest risk of ORC, which has been observed in other cohort studies. Data from the Women's Health Initiative showed a greater risk for colorectal cancer in postmenopausal MUNW females than MHNW (OR 1.79, 95% CI 1.02–3.12).^10^ Other reports have observed associations of cancer with the metabolically unhealthy phenotypes, either with^11^, ^12^, ^13^, ^34^ or independent from obesity.^14^, ^15^, ^16^, ^17^, ^18^, ^19^, ^20^, ^21^ A recent pooled analysis of 797,193 participants in the Metabolic syndrome and Cancer (Me‐Can) project 2.0 observed statistically significant higher risks of ORC with all abnormal metabolic obesity phenotypes, with metabolically unhealthy obese individuals having the greatest risk ([OR (95% CI): 1.91 (1.74–2.09) and 1.43 (1.35–1.51), in men and women, respectively]).^35^ This study concluded that metabolic dysfunction further increases ORC risk and may be a useful target for prevention in addition to obesity. Additionally, using UK Biobank data on 390,575 individuals, Cao et al.^19^ observed that MUO individuals had a higher risk of 10 ORCs, in contrast to MHO individuals that had a higher risk of only five ORCs. Together, these observed associations suggest that metabolic dysfunction may be a better metric to risk stratify individuals for their risk of cancer, rather than obesity alone.^6^, ^22^, ^23^

The majority of studies evaluating metabolic obesity phenotypes and ORC risk used MetS criteria to identify metabolically unhealthy participants. Some used the NCEP ATP III definition of MetS (≥3 MetS criteria), and using this strict definition, the metabolically unhealthy phenotypes were associated with a higher risk of colorectal, pancreatic, breast and thyroid cancers.^10^, ^14^, ^18^, ^21^, ^29^ In our study, we performed analyses using both the broader (≥1 MetS criteria) and clinical (≥3 MetS criteria) definitions of metabolic dysfunction and observed that only the broader definition was associated with a higher risk of ORC. We ran additional analyses categorising all participants without MetS as metabolically healthy (i.e. the reference group phenotype included participants with 1 or 2 MetS criteria) to further understand the clinical relevance of these findings. As expected, the strength of the estimates for each abnormal metabolic obesity phenotype diminished and none were associated with ORC risk. Although MetS is a clinically useful tool, these results suggest that participants with mild metabolic dysfunction that may not necessarily qualify as having MetS (i.e. 1 or 2 abnormal parameters compared to 3 or more) may still be at a higher risk of ORC compared to metabolically healthy individuals. We also evaluated dysfunction in each metabolic parameter individually to determine which parameter was driving the increased risk of ORC. We observed that participants who only had hypertension or only central obesity had a 3.7‐ and 2.2‐times higher risk of ORC, respectively, compared to MHNW participants. We also observed that all MUNW participants with hyperglycaemia had a 63% higher ORC risk compared with MHNW participants.

Similar to our analysis, Park et al.^18^ evaluated MetS, as well as each individual component of MetS, with breast cancer risk in an East Asian cohort. Their results were largely similar to ours with increased risk for MUNW compared with MHNW phenotype.^18^ Additionally, they observed stronger associations of breast cancer with MetS, hypertension, hyperglycaemia, abdominal obesity and low‐HDL cholesterol in the MUO phenotype compared with the MHO phenotype. Differences in diet, lifestyle and physical activity between our study population and theirs and their focus only on breast cancer may explain these results. Moore et al.^15^ analysed presence of hyperglycaemia as a metric of metabolic dysfunction in a cohort of 3763 participants from the Framingham Heart Study and observed that cancer risk may be lower in MHO individuals compared to those who are metabolically unhealthy. Similar to this study, we observed in our data set that MUNW participants with hyperglycaemia had a statistically significant higher ORC risk. Three studies used C‐peptide or the homeostatic model assessment for insulin resistance (HOMA‐IR) to define metabolic dysfunction and observed that individuals with hyperinsulinemia had a higher risk of cancer,^6^, ^13^, ^22^ suggesting altered glucose metabolism may be an important risk factor for ORC.

The more recent consensus definition for metabolic syndrome criteria (incorporating the International Diabetes Federation and American Heart Association and the National Heart, Lung, and Blood Institute definitions) proposed by Kassi et al.^36^ does not have a specific cut point for waist circumference, but rather suggests using population and country‐specific definitions as some ethnic groups may be susceptible to MetS below these cut points.^37^ The NCEP‐ATP III cut points were defined according to the 1998 National Institutes of Health obesity clinical guidelines and approximate the upper quartile of the US population.^38^ As NHANES lacks detailed race and ethnicity data, the NCEP‐ATP III cut points were used for all participants. Owing to this, and because of the high correlation of waist circumference with BMI, we performed a sensitivity analysis excluding waist circumference in the metabolic health definition. These results were similar to our overall results and did not change after adjusting for waist circumference (Table S2). Other prior studies used the MetS definition, excluding waist circumference (to separate other metabolic parameters from obesity) and observed stronger associations with the metabolically unhealthy phenotypes.^11^, ^19^, ^20^, ^23^, ^39^ This suggests that other components of metabolic dysfunction, such as hyperglycaemia, hypertension and dyslipidaemia, may be driving the higher risk of ORC in the MUNW phenotype. Finally, some studies suggest that transitioning from a metabolically unhealthy to a metabolically healthy phenotype in overweight or obese individuals may be possible through weight loss.^40^ Indeed, bariatric surgical treatment has been shown to decrease ORC risk,^41^ suggesting potential benefits of weight reduction and mitigation of central adiposity.

This study has some limitations. First, NHANES is cross‐sectional, therefore we are unable to determine the temporality between metabolic dysfunction and ORC risk. It is possible that participants transitioned from being metabolically healthy to unhealthy, or experienced weight loss after their cancer diagnosis due to changes in physical activity, diet and cancer treatment‐related mechanisms.^42^, ^43^, ^44^, ^45^, ^46^ Second, there is a possibility of misclassification bias and underreported ORC as cancers in NHANES are self‐reported. Also, less than perfect measurement of biomarkers used to determine metabolic dysfunction could also result in misclassification. However, these errors are expected to be non‐differential and therefore will only bias the results toward the null. There is a possibility that NHANES participants with cancer have less aggressive or earlier stage cancers, thus this cancer population may not be representative of all incident cancers. Lastly, these data include a small sample of heterogeneous ORC cases (*n* = 528) that precluded the assessment of individual cancer types, stratified analysis by sex and additionally resulted in lower sample size and power in certain phenotype groups. We also had to exclude some ORCs, including oesophageal adenocarcinoma, gastric cardia, meningioma and multiple myeloma, due to the lack of histological data in NHANES. However, there were few cases of these rarer cancer types in NHANES [*N* = 107(0.11%)] and is therefore unlikely to impact our results. Despite these limitations, we believe that this study represents an important first step in examining the hypothesis that metabolic obesity phenotypes are associated with cancer, and that they may be a better tool to assess an individual's risk of cancer compared to obesity as ascertained by BMI alone. A great strength of this study is that the NHANES data are nationally representative, making these results generalisable to the U.S. civilian non‐institutionalized population. Using these data, we were able to estimate the percent of individuals in each phenotype group among cancer‐free and obesity‐related cancer populations. Furthermore, these data were collected through detailed surveys and extensive physical and laboratory examinations, allowing thorough investigation of metabolic obesity phenotype and the risk of multiple ORCs.

## 5 CONCLUSIONS

The results from this study suggest that MUNW individuals may have a higher ORC risk compared to MHNW individuals. As such, the monitoring and management of metabolic dysfunction parameters may be crucial in cancer prevention. Additionally, tools such as metabolic obesity phenotypes may be a better method of risk stratifying individuals for ORC than obesity measures alone. However, as there is currently no universally accepted definition of metabolic obesity phenotypes,^47^ further research is needed to determine what phenotypes are associated with the highest risk of cardiometabolic diseases, including cancer. Future directions of this research are to further examine the association between metabolic obesity phenotypes by cancer site, and to investigate metabolic phenotypes in relation to mortality outcomes among cancer survivors.

## AUTHOR CONTRIBUTIONS

**Maci Winn:** Conceptualization (lead); formal analysis (lead); investigation (lead); methodology (lead); project administration (lead); visualization (lead); writing – original draft (lead); writing – review and editing (lead). **Prasoona Karra:** Conceptualization (supporting); investigation (supporting); methodology (supporting); visualization (supporting); writing – review and editing (supporting). **Heinz Freisling:** Methodology (supporting); writing – review and editing (supporting). **Marc J Gunter:** Conceptualization (supporting); methodology (supporting); writing – review and editing (supporting). **Benjamin Haaland:** Formal analysis (supporting); methodology (supporting); visualization (supporting); writing – review and editing (supporting). **Michelle Litchman:** Methodology (supporting); writing – review and editing (supporting). **Jennifer A Doherty:** Methodology (supporting); writing – review and editing (supporting). **Mary C Playdon:** Conceptualization (equal); investigation (equal); methodology (equal); project administration (equal); supervision (equal); visualization (equal); writing – original draft (equal); writing – review and editing (equal). **Sheetal Hardikar:** Conceptualization (equal); formal analysis (equal); funding acquisition (equal); investigation (equal); methodology (equal); project administration (equal); supervision (equal); visualization (equal); writing – original draft (equal); writing – review and editing (equal).

## FUNDING INFORMATION

This study was supported by the following grants from the National Institute of Health: NCI K07 CA222060 (S. Hardikar), NCI R00 CA218694 (M. Playdon). Research reported herein utilized the Cancer Biostatistics Shared Resource at the Huntsman Cancer Institute, University of Utah and was supported by NCI P30 CA042014. The content does not necessarily represent the official views of the NIH.

## CONFLICT OF INTEREST STATEMENT

The authors declare no conflicts of interest.

## ETHICAL APPROVAL

All participants in the National Health and Nutrition Examination Survey (NHANES), a population‐based study conducted yearly in the United States, provide written informed consent, and study protocols are approved by the institutional review boards at the Centers for Disease Control and Prevention. These data are made publicly available and are therefore exempt from individual institutional review.

## IARC DISCLAIMER

Where authors are identified as personnel of the International Agency for Research on Cancer/World Health Organization, the authors alone are responsible for the views expressed in this article and they do not necessarily represent the decisions, policy or views of the International Agency for Research on Cancer/World Health Organization.

## Supporting information


Appendix S1

## Data Availability

Some or all data generated or analysed during this study are included in this published article or in the data repositories listed in References. URL: https://wwwn.cdc.gov/nchs/nhanes/Default.aspx.
