# Supplementary material for: Metabolic obesity phenotypes and obesity‐related cancer risk in the National Health and Nutrition Examination Survey
Source: Endocrinol Diabetes Metab. 2023 Jun 5;6(4):e433. doi: 10.1002/edm2.433 (PMC10335619; doi:10.1002/edm2.433)
Supplement: Supplementary file 1 — Appendix S1 [file EDM2-6-e433-s001.docx]

**SUPPLEMENTARY TABLES**

**Supplementary Table 1: Demographic information for National Health and Nutrition Examination Survey (NHANES) participants by metabolic obesity phenotype (N = 19,500)** ***^a^*.**

|  | **MHNW, n =2,605** | | **MUNW, n = 3,585** | | **MHO, n = 928** | | **MUO, n = 12,100** | |
| --- | --- | --- | --- | --- | --- | --- | --- | --- |
|  | *n/mean* | *%/SE* | *n/mean* | *%/SE* | *n/mean* | *%/SE* | *n/mean* | *%/SE* |
| **Age in years***^b^* |  |  |  |  |  |  |  |  |
| <50 | 2,232 | 85.1 | 1,705 | 53.6 | 791 | 86.6 | 6,002 | 55.7 |
| 50-59 | 201 | 9.8 | 518 | 17.9 | 74 | 8.8 | 2,031 | 19.6 |
| 60-69 | 106 | 3.6 | 581 | 13.2 | 38 | 2.9 | 2,197 | 14.3 |
| 70-79 | 44 | 1.0 | 414 | 8.8 | 12 | 1.1 | 1,271 | 7.3 |
| ≥80 | 22 | 0.5 | 367 | 6.6 | 13 | 0.6 | 599 | 3.1 |
| **Sex** |  |  |  |  |  |  |  |  |
| Female | 1,457 | 60.7 | 1,742 | 51.2 | 292 | 30.0 | 6,233 | 50.2 |
| Male | 1,148 | 39.3 | 1,843 | 48.8 | 636 | 70.0 | 5,867 | 49.8 |
| **Race/ethnicity***^c^* |  |  |  |  |  |  |  |  |
| White (non-Hispanic) | 1,132 | 69.9 | 1,658 | 68.3 | 337 | 60.6 | 5,060 | 67.0 |
| Black (non-Hispanic) | 545 | 10.3 | 569 | 8.9 | 248 | 15.7 | 2,611 | 12.6 |
| MA/ Hispanic | 578 | 10.9 | 808 | 10.7 | 282 | 18.2 | 3,780 | 15.6 |
| Other | 350 | 8.9 | 550 | 12.1 | 61 | 5.5 | 649 | 4.8 |
| **Income***^e^* |  |  |  |  |  |  |  |  |
| <$35,000 | 1,121 | 34.3 | 1,807 | 41.5 | 386 | 31.9 | 5,723 | 37.0 |
| $35,000-$75,000 | 700 | 28.8 | 958 | 30.7 | 269 | 29.7 | 3,667 | 33.7 |
| >$75,000 | 415 | 19.9 | 459 | 16.5 | 152 | 20.9 | 1,495 | 17.7 |
| **Education***^e^* |  |  |  |  |  |  |  |  |
| <High School | 598 | 15.2 | 1,105 | 20.8 | 214 | 14.9 | 3,664 | 20.1 |
| High School | 572 | 20.2 | 876 | 25.2 | 213 | 22.1 | 2,888 | 25.0 |
| >High School | 1,434 | 64.6 | 1,595 | 53.8 | 500 | 62.8 | 5,540 | 54.8 |
| **Smoking***^e^* |  |  |  |  |  |  |  |  |
| Never | 1,337 | 55.8 | 1,673 | 46.9 | 504 | 55.7 | 6,337 | 52.6 |
| Former | 304 | 14.4 | 753 | 21.6 | 146 | 18.6 | 3,125 | 26.6 |
| Current | 497 | 22.4 | 908 | 28.0 | 167 | 20.7 | 2,208 | 19.2 |
| **Body mass index (kg/m^2^)** *^e^* | 21.8 | 0.1 | 22.4 | 0.04 | 27.3 | 0.09 | 32.1 | 0.09 |
| **Total daily caloric intake***^e^* | 2,238 | 22 | 2,107 | 21 | 2,413 | 38 | 2,119 | 11 |
| **Physical activity***^e^* |  |  |  |  |  |  |  |  |
| No/low | 657 | 21.7 | 1,299 | 30.7 | 224 | 20.2 | 4,494 | 31.8 |
| Moderate/  vigorous | 1,936 | 77.9 | 2,228 | 67.8 | 698 | 79.2 | 7,411 | 66.9 |
| **Sedentary***^e^* |  |  |  |  |  |  |  |  |
| < 5 hours | 1,055 | 38.9 | 1,424 | 39.2 | 366 | 38.0 | 5,302 | 46.1 |
| ≥ 5 hours | 1,549 | 61.0 | 2,159 | 60.7 | 562 | 62.0 | 6,795 | 53.9 |
| **Alcohol Use***^e^* |  |  |  |  |  |  |  |  |
| No | 460 | 15.9 | 835 | 20.6 | 133 | 11.4 | 3,016 | 21.6 |
| Yes | 1,333 | 61.7 | 1,912 | 59.5 | 545 | 66.6 | 6,848 | 61.6 |
| **Menopausal Status***^de^* |  |  |  |  |  |  |  |  |
| Premenopausal | 1,024 | 69.3 | 582 | 38.4 | 187 | 68.0 | 2,310 | 39.9 |
| Postmenopausal | 302 | 22.9 | 1,030 | 55.1 | 68 | 21.3 | 3,427 | 53.0 |
| **HRT use***^de^* |  |  |  |  |  |  |  |  |
| No | 959 | 73.4 | 1,065 | 64.5 | 182 | 70.3 | 4,180 | 67.3 |
| Yes | 128 | 11.8 | 415 | 25.4 | 31 | 11.2 | 1,302 | 23.5 |

*^a^Sample sizes reflect the actual selected sample numbers. Means and proportions were weighted according to the NHANES analytic guidelines.*

*^b^Participants with age ≥ 85 are recorded as age = 85 in the NHANES datasets.*

*^c^The “other” race category in NHANES includes non-Hispanic Asian and all non-Hispanic persons that reported races other than Black, Asian, or White.*

*^d^Among* *female study participants only.*

*^e^Data was missing for the following: BMI: 1.5%, Smoking: 6.6%, Alcohol: 21.9%, Physical Activity: 1.6%, Sedentary Behavior: 0.03%, Total daily caloric intake: 3.5%, Menopausal Status: 8.4%, HRT: 15.3%, Income: 10.7%, Education: 0.1%.*

*Abbreviations: MA, Mexican American; HRT, hormone replacement therapy.*

**Supplementary Table 2: Adjusted odds ratios (OR) and 95% confidence intervals (CI) for obesity-related cancer (ORC) by each metabolic obesity phenotype, excluding waist circumference from the metabolic syndrome criteria, in National Health and Nutrition Examination Survey participants (N = 19,500).**

|  | **Cancer-free** | | **ORC Cases** | | *Fully Adjusted^a^* | |
| --- | --- | --- | --- | --- | --- | --- |
| **Metabolic Obesity Phenotype** | **N**  *(actual)* | **%**  *(weighted)* | **N**  *(actual)* | **%**  *(weighted)* | **OR** | **95% CI** |
| **Metabolic dysfunction defined as one or more MetS criteria (MODEL A)*^b^*** | | | | | | |
| MHNW (Ref) | 2,684 | 16.0 | 26 | 1.5 | REF | REF |
| MUNW | 3,353 | 16.7 | 127 | 2.4 | 2.00 | 1.21-3.30 |
| MHO | 2,252 | 12.9 | 35 | 1.7 | 1.48 | 0.83-2.65 |
| MUO | 10,411 | 53.3 | 330 | 2.0 | 1.38 | 0.85-2.24 |
| **Metabolic dysfunction defined as one or two MetS criteria (MODEL B)*^c^*** | | | | | | |
| MHNW (Ref) | 2,684 | 19.4 | 26 | 9.5 | REF | REF |
| MUNW | 2,888 | 17.8 | 104 | 31.2 | 2.01 | 1.20-3.37 |
| MHO | 2,252 | 15.6 | 35 | 11.6 | 1.50 | 0.83-2.71 |
| MUO | 7,228 | 45.7 | 191 | 44.6 | 1.20 | 0.73-1.96 |
| **Metabolic dysfunction defined as the clinical definition of MetS (≥3 criteria) (MODEL C)*^d^*** | | | | | | |
| MHNW (Ref) | 2,684 | 33.5 | 26 | 15.1 | REF | REF |
| MUNW | 465 | 4.3 | 23 | 9.5 | 1.90 | 0.94-3.84 |
| MHO | 2,252 | 26.9 | 35 | 18.3 | 1.56 | 0.88-2.77 |
| MUO | 3,183 | 32.8 | 139 | 52.3 | 2.00 | 1.12-3.59 |

*^a^Adjusted for age, sex, race/ethnicity, education level, annual household income, smoking status, alcohol use, daily hours sedentary, weekly physical activity level, daily calorie intake, and survey year.*

*^b^MODEL A: Cancer-free, n=18,972; ORC cases, n=528.*

*^c^MODEL B: Cancer-free, n=* *12,434; ORC cases, n=259 (excludes participants with ≥3 MetS criteria).*

*^d^MODEL C: Cancer-free, n=* *10,315; ORC cases, n=307 (excludes participants with 1-2 MetS criteria).*

*Abbreviations: MUNW, metabolically unhealthy normal weight; MHO, metabolically healthy overweight/obese; MUO, metabolically unhealthy overweight/obese.*

**Supplementary Table 3: Adjusted odds ratios (OR) and 95% confidence intervals (CI) for obesity-related cancer (ORC) by each metabolic obesity phenotype in female National Health and Nutrition Examination Survey participants (N = 9,876).**

|  | **Cancer-free,**  **n = 9,418** | | **ORC Cases,**  **n = 458** | | *Fully Adjusted^a^* | |
| --- | --- | --- | --- | --- | --- | --- |
| **Metabolic Obesity Phenotype** | **N**  *(actual)* | **%**  *(weighted)* | **N**  *(actual)* | **%**  *(weighted)* | **OR** | **95% CI** |
| **Metabolic dysfunction defined as one or more MetS criteria (MODEL A)*^b^*** | | | | | | |
| MHNW (Ref) | 1,439 | 18.4 | 18 | 5.5 | REF | REF |
| MUNW | 1,624 | 17.3 | 118 | 29.2 | 2.40 | 1.33-4.36 |
| MHO | 286 | 3.1 | 6 | 1.7 | 1.92 | 0.57-6.44 |
| MUO | 5,924 | 60.0 | 309 | 61.6 | 1.57 | 0.88-2.79 |
| **Metabolic dysfunction defined as one or two MetS criteria (MODEL B)*^c^*** | | | | | | |
| MHNW (Ref) | 1,439 | 27.2 | 18 | 9.8 | REF | REF |
| MUNW | 1,293 | 20.7 | 89 | 42.7 | 2.81 | 1.51-5.21 |
| MHO | 286 | 4.6 | 6 | 3.0 | 1.93 | 0.56-6.64 |
| MUO | 2,925 | 45.7 | 100 | 40.7 | 1.53 | 0.82-2.85 |
| **Metabolic dysfunction defined as the clinical definition of MetS (≥3 criteria) (MODEL C)*^d^*** | | | | | | |
| MHNW (Ref) | 1,439 | 33.5 | 18 | 10.2 | REF | REF |
| MUNW | 331 | 5.8 | 29 | 10.2 | 1.57 | 0.75-3.31 |
| MHO | 286 | 5.6 | 6 | 3.1 | 1.86 | 0.54-6.37 |
| MUO | 2,999 | 52.8 | 209 | 72.7 | 1.67 | 0.89-3.14 |

*^a^Adjusted for age, race/ethnicity, education level, annual household income, smoking status, alcohol use, daily hours sedentary, weekly physical activity level, daily calorie intake, survey year, hormone replacement therapy use, and menopausal status.*

*^b^MODEL A: Cancer-free, n=9,418; ORC cases, n=458.*

*^c^MODEL B: Cancer-free, n=* *6,088; ORC cases, n=220 (excludes participants with ≥3 MetS criteria).*

*^d^MODEL C: Cancer-free, n=* *5,200; ORC cases, n=269 (excludes participants with 1-2 MetS criteria).*

*Abbreviations: MUNW, metabolically unhealthy normal weight; MHO, metabolically healthy overweight/obese; MUO, metabolically unhealthy overweight/obese.*

**Supplementary Table 4: Adjusted odds ratios (OR) and 95% confidence intervals (CI) for obesity-related cancer (ORC) by each metabolic obesity phenotype, excluding overweight participants, in National Health and Nutrition Examination Survey participants (N = 13,119).**

|  | **Cancer-free,** | | **ORC Cases,** | | *Age and Sex Adjusted* | | *Fully Adjusted^a^* | | |
| --- | --- | --- | --- | --- | --- | --- | --- | --- | --- |
|  |  | |  | |  |  |  |  |  |
| **Metabolic Obesity Phenotype**^b^ | **N**  *(actual)* | **%**  *(weighted)* | **N**  *(actual)* | **%**  *(weighted)* | **OR** | **95% CI** | **OR** | **95% CI** | |
| **Metabolic dysfunction defined as one or more MetS criteria (MODEL A)*^c^*** | | | | | | | | |  |
| MHNW (Ref) | 2,584 | 22.6 | 21 | 7.7 | REF | REF | REF | REF | |
| MUNW | 3,453 | 25.9 | 132 | 39.7 | 2.23 | 1.24-3.99 | 2.23 | 1.25-3.98 | |
| MHO | 94 | 0.7 | N/A | N/A | N/A | N/A | N/A | N/A | |
| MUO | 6,348 | 49.0 | 205 | 49.6 | 1.71 | 0.99-2.95 | 1.67 | 0.98-2.86 | |
| **Metabolic dysfunction defined as one or two MetS criteria (MODEL B)*^d^*** | | | | | | | | | |
| MHNW (Ref) | 2,584 | 33.8 | 21 | 13.8 | REF | REF | REF | REF | |
| MUNW | 2,871 | 32.8 | 101 | 57.3 | 2.48 | 1.35-4.54 | 2.46 | 1.34-4.51 | |
| MHO | 94 | 1.1 | N/A | N/A | N/A | N/A | N/A | N/A | |
| MUO | 2,524 | 29.7 | 49 | 23.4 | 1.35 | 0.73-2.51 | 1.40 | 0.74-2.62 | |
| **Metabolic dysfunction defined as the clinical definition of MetS (≥3 criteria) (MODEL C)*^e^*** | | | | | | | | | |
| MHNW (Ref) | 2,584 | 38.8 | 21 | 14.1 | REF | REF | REF | REF | |
| MUNW | 582 | 6.9 | 31 | 13.8 | 1.55 | 0.71-3.40 | 1.66 | 0.77-3.59 | |
| MHO | 94 | 1.2 | N/A | N/A | N/A | N/A | N/A | N/A | |
| MUO | 3,824 | 50.1 | 156 | 66.5 | 1.92 | 0.99-3.69 | 1.94 | 1.02-3.67 | |

*^a^Adjusted for age, sex, race/ethnicity, education level, annual household income, smoking status, alcohol use, daily hours sedentary, weekly physical activity level, daily calorie intake, and survey year.*

*^b^Weight component of the metabolic obesity phenotype was determined using body mass index as <25 kg/m^2^ = normal weight, and ≥30 kg/m^2^ = obese.*

*^c^MODEL A: Cancer-free, n=* *12,751; ORC cases, n=368.*

*^d^MODEL B: Cancer-free, n=* *8,345; ORC cases, n=161 (excludes participants with ≥3 MetS criteria).*

*^e^MODEL C: Cancer-free, n=* *7,356; ORC cases, n=218 (excludes participants with 1-2 MetS criteria).*

*Abbreviations: MUNW, metabolically unhealthy normal weight; MHO, metabolically healthy overweight/obese; MUO, metabolically unhealthy overweight/obese.*

**Supplementary Table 5: Complete case analysis with adjusted odds ratios (OR) and 95% confidence intervals (CI) for obesity-related cancer (ORC) by each metabolic obesity phenotype in National Health and Nutrition Examination Survey participants (N=** **16,811).**

|  | **Cancer-free,**  **n = 16,373** | | **ORC Cases,**  **n = 438** | | *Fully Adjusted^a^* | |
| --- | --- | --- | --- | --- | --- | --- |
| **Metabolic Obesity Phenotype** | **N**  *(actual)* | **%**  *(weighted)* | **N**  *(actual)* | **%**  *(weighted)* | **OR** | **95% CI** |
| **Metabolic dysfunction defined as one or more MetS criteria (MODEL A)*^b^*** | | | | | | |
| MHNW (Ref) | 2,176 | 14.9 | 17 | 5.4 | REF | REF |
| MUNW | 3,063 | 17.9 | 114 | 30.0 | 2.33 | 1.26-4.31 |
| MHO | 758 | 5.0 | 6 | 1.7 | 1.73 | 0.51-5.93 |
| MUO | 10,376 | 62.2 | 301 | 62.9 | 1.55 | 0.84-2.83 |
| **Metabolic dysfunction defined as one or two MetS criteria (MODEL B)*^c^*** | | | | | | |
| MHNW (Ref) | 2,176 | 22.8 | 17 | 10.8 | REF | REF |
| MUNW | 2,520 | 22.9 | 85 | 47.0 | 2.63 | 1.40-4.94 |
| MHO | 758 | 7.7 | 6 | 3.5 | 1.78 | 0.50-6.34 |
| MUO | 4,935 | 46.7 | 84 | 38.7 | 1.34 | 0.69-2.59 |
| **Metabolic dysfunction defined as the clinical definition of MetS (≥3 criteria) (MODEL C)*^d^*** | | | | | | |
| MHNW (Ref) | 2,176 | 27.4 | 17 | 9.5 | REF | REF |
| MUNW | 543 | 5.3 | 29 | 11.0 | 1.62 | 0.75-3.52 |
| MHO | 758 | 9.3 | 6 | 3.1 | 1.65 | 0.48-5.68 |
| MUO | 5,441 | 58.1 | 217 | 76.3 | 1.65 | 0.84-3.26 |

*^a^Adjusted for age, sex, race/ethnicity, education level, annual household income, smoking status, alcohol use, daily hours sedentary, weekly physical activity level, daily calorie intake, and survey year.*

*^b^MODEL A: Cancer-free, n=* *16,373; ORC cases, n=438.*

*^c^MODEL B: Cancer-free, n=* *10,389; ORC cases, n=192 (excludes participants with ≥3 MetS criteria).*

*^d^MODEL C: Cancer-free, n=* *8,918; ORC cases, n=269 (excludes participants with 1-2 MetS criteria).*

*Abbreviations: MUNW, metabolically unhealthy normal weight; MHO, metabolically healthy overweight/obese; MUO, metabolically unhealthy overweight/obese.*
